# Tongue microbiome in children with autism spectrum disorder

**DOI:** 10.1080/20002297.2021.1936434

**Published:** 2021-06-22

**Authors:** Ahmed Abdulhaq, Esam Halboub, Husham E. Homeida, Vinod Kumar Basode, Ahmad Hassn Ghzwani, Khalid Ammash Zain, Divyashri Baraniya, Tsute Chen, Nezar Noor Al-Hebshi

**Affiliations:** a Department of Medical Laboratory Technology, College of Applied Medical Sciences, Jazan University, Jazan, Kingdom of Saudi Arabia; b Department of Maxillofacial Surgery and Diagnostic Sciences, College of Dentistry, Jazan University, Jazan, Kingdom of Saudi Arabia; c Department of Oral Medicine, Oral Pathology and Oral Radiology, Faculty of Dentistry, Sana’a University, Yemen; d Medical Research Centre, Jazan University, Jazan, Kingdom of Saudi Arabia; e Department of Oral Health Sciences, Oral Microbiome Research Laboratory, Maurice H. Kornberg School of Dentistry, Temple University, Philadelphia, PA, USA; f Department of Microbiology, Forsyth Institute, Cambridge, MA, USA

**Keywords:** Autism spectrum disorder, bacteria, high-throughput nucleotide sequencing, mouth, microbiota, tongue

## Abstract

**Background**: A few recent studies have characterized the salivary microbiome in association with Autism Spectrum Disorder (ASD). Here, we sought to assess if there is an association between the tongue microbiome and ASD.

**Methods**: Tongue scrapping samples were obtained from 25 children with ASD and 38 neurotypical controls. The samples were sequenced for the *16S rRNA* gene (V1-V3) and the resultant high-quality reads were assigned to the species-level using our previously described BLASTn-based algorithm. Downstream analyses of microbial profiles were conducted using QIIME, LEfSe, and R.

**Results**: Independent of grouping, *Prevotella, Streptococcus, Leptotrichia, Veillonella, Haemophilus* and *Rothia* accounted for > 60% of the average microbiome. *Haemophilus parainfluenzae, Rothia mucilaginosa, Prevotella melaninogenica* and *Neisseria flavescens/subflava* were the most abundant species. Species richness and diversity did not significantly differ between the study groups. Thirteen species and three genera were differentially abundant between the two groups, e.g. enrichment of *Actinomyces odontolyticus* and *Actinomyces lingnae* and depletion of *Campylobacter concisus* and *Streptococcus vestibularis* in the ASD group. However, none of them withstood adjustment for multiple comparisons.

**Conclusion**: The tongue microbiome of children with ASD was not significantly different from that of healthy control children, which is largely consistent with results from the literature.

## Introduction

Autism Spectrum Disorders (ASD) encompasses a group of disorders characterized by early-onset social communication deficits and repetitive sensory-motor behaviors. The disorders have a predominantly genetic component [1], as 74 to 93% of ASD have been identified as heritable [2], but other risk factors have been reported [3]. ASD ranges from very mild to severe, and many individuals require lifelong support [1]. WHO estimated the global prevalence of ASD to be 1% [4]. ASD is associated with a plethora of coexisting conditions, including intellectual disability (IQ < 70%) which reported in 15 to 65% of studied samples [5], sleeping disorders and others [6]. Early changes in brain development and neural reorganization have been identified as mechanisms in ASD development pathways, however, due to unavailability of reliable biomarkers, the diagnosis is made mainly on the basis of behavior [7,8].

Several studies have linked the gut microbiome to ASD [9–13] substantiating evidence for the so-called brain-gut-microbiome axis [14,15]. The latter refers, to the interactions between the central nervous system and gastrointestinal system, including its resident microbial community. These interactions are believed to play a role in behavioral and neurodegenerative diseases. A recent systematic review summarized the differences in gut microbiome of ASD and healthy individuals as a decrease in the genera *Bifidobacterium, Blautia, Dialister, Prevotella, Veillonella*, and *Turicibacter*, in contrast to an increase in *Lactobacillus, Bacteroides, Desulfovibrio*, and *Clostridium* [16]. Furthermore, interventional studies using fecal microbiota transplants, prebiotics and probiotics to modulate the gut microbiome in patients with psychiatric disorders including ASD have found promising results [16,17].

The oral cavity is a home to the second most diverse microbial community after that of the gut [18], and there is emerging evidence to suggest presence of a microbial oral-brain axis [19]. Indeed, studies have found an association between the oral microbiome and neurological diseases including Alzheimer’s disease [20] and Parkinson’s disease [21]. Similarly, a few recent studies have identified an altered oral microbiome in association with ASD [22–24], although the differences were minimal. All these studies used saliva as a sample for microbiome analysis. However, saliva contains a mixture of microorganism from different sites of the oral cavity and is thus habitat non-specific. Oral diseases are associated with microbial alterations in particular habitats, for example subgingival plaque in periodontitis, supragingival plaque in dental caries and dorsum of the tongue in halitosis. The latter in particular provides a large and rough surface for colonization of a unique and dense microbial community [25,26] that has higher chance to interact with the host compared to microbial communities colonizing smooth mucosal surfaces. Indeed, the tongue microbiome has been recently implicated in regulation of blood pressure [27]. The objective of this study was therefore to assess the potential association between tongue microbiome and ASD.

## Material and methods

### Study design and population

This cross-sectional study was conducted during the 2018/2019 academic year in Jazan city, Jazan, Saudi Arabia. The participants with ASD were recruited from public schools officially assigned to enroll ASD patients in certain classes with the aim of integrating them with the healthy students. The ASD students are accepted based on formal reports issued by the Hope Hospital and Mental Health, a specialized center for diagnosis and treatment of behavioral and psychological diseases. The hospital applies the Diagnostic and Statistical Manual of Mental Disorders criteria (DSM-5) [28] for ASD diagnosis. Neurotypical (healthy) controls were recruited from among the healthy students attending the same schools. A structured questionnaire was completed by the parent to collect demographic data and relevant medical history. Clinical examination was done in an office chair under natural light. Bleeding on probing and dental caries using the Decay, Missing Filling index for Teeth (DMFT) were assessed using disposable dental examination sets. Participants who had bad oral health (gingivitis: gingival bleeding on probing in more than 10 of the sites) [29], and/or had a history of using antimicrobial and/or steroids within the last 3 months were excluded.

The Scientific Research Ethics Committee, Jazan University, approved the study (REC39/3–463). The study complied with the Helsinki Declaration on medical research involving human subjects. Written informed consents were obtained from at least one parent of each child.

### Tongue scraping and DNA extraction

Tongue scraping samples were collected in the morning between 9 am and 12 pm after completing the clinical examination by at least half an hour to ensure that the participants did not eat or drink prior to sample collection. The participants were not given specific instruction about performing or refraining from oral hygiene and tongue brushing. Each participant was asked to protrude his/her tongue forward before it was stabilized by the examiner by holding the tip with a piece of sterile gauze. The dorsal surface of the tongue was dried with another piece of sterile gauze and a sterilized wooden spatula was used to scrap the surface with overlapping strokes starting posteriorly all the way to the tip. A sterile paper point was used to transfer the collected scraping into a sterile Eppendorf tube containing 600 μl sterile, molecular-grade Tris-EDTA buffer (pH 8.0) and stored at − 20°C.

At the time of DNA extraction, the samples were thawed, vigorously vortexed, and centrifuged at 14,000 rpm for 1 minute (Micro 120, Hettich Zentrifuge, Germany). After decanting the supernatant, the pellet was washed with 500 μl phosphate-buffered saline, suspended in 180 μl of lysozyme solution (20 mg/ml), and incubated overnight at 37°C. The PureLink™ Genomic DNA Mini Kit (Invitrogen, USA) was used for DNA extraction from the digests according to the manufacturer’s instructions, and using an elution volume of 100 μl. A Jenway Genova Nano 3-in-1 Spectrophotmeter (Jenway®, UK) was used to assess the quantity of DNA. The resultant extracts were then stored at − 20°C for subsequent analysis.

### 16S sequencing and bioinformatic analysis

Preparation of library and sequencing of the *16S rRNA* gene were performed, as described elsewhere [30], at the Australian Center for Ecogenomics (Brisbane, Australia). Briefly, the V1-3 region was amplified using the degenerate primers 27FYM [31] and 519 R [32] and the generated amplicons (~ 520 bp) were purified, and tagged with 8-base barcodes. The resultant libraries were pooled in equimolar concentrations and sequenced on a MiSeq (Illumina, USA) using the Illumina’s V3 2 × 300 bp chemistry. The minimum sequencing depth was set to 30,000 reads per sample

The raw paired reads were merged with PEAR [33], and trimmed and quality-controlled with mothur [34] as detailed previously [35]. The resultant high quality, merged reads were then classified using our BLASTn-based, species-level taxonomy assignment algorithm as described in details elsewhere [35,36]. In brief, the algorithm searches individual reads at alignment coverage and % identity more than 98% against four 16S rRNA reference databases ranked according to biological relevance. Each read was then assigned taxonomy of the hit sequence with the highest % identity and bit score belonging to the highest priority reference set. Unassigned reads were clustered *de novo* into operational taxonomical units (OTUs); OTUs with less than 100 sequences were filtered out, and the remaining were considered as potentially novel taxa.

The Quantitative Insights into Microbial Ecology (QIIME) software package version 1.9.1 [37] was used for downstream analysis of microbial profiles including generation of taxonomy plots/tables and rarefaction curves, calculation of species richness, coverage, alpha diversity indices and beta diversity distance matrices. Principle coordinate analysis (PCA) was performed with statistical analysis of taxonomic and functional profiles (STAMP) [38]. Differentially abundant taxa were identified with linear discriminant analysis (LDA) effect size (LEfSe) [39]. The results of the latter were adjusted for false discovery rates (FDR) using the Benjamini-Hochberg method [40].

## Results

### Characterizations of the study sample

The study sample comprised 63 children: 25 with ASD (16 were males) and 38 neurotypical controls (18 were males). There were no significant differences between the two groups in age or caries status except that the ASD group had a significantly higher mean of filled primary teeth (Table 1).

**Table 1.** Demographic data and clinical features of the study groups

Variable	Categories	Group		P value*
		Autism (n = 25)	Control (n = 38)	
Age (year)		9.24 ± 1.96	10.03 ± 1.48	0.075
Gender	Males	16 (64)	18 (47.4)	0.150
	Females	9 (36)	20 (52.6)	
decay (d)		2.36 ± 2.18	2.53 ± 3.1	0.803
missing (m)		0.16 ± 0.8	0.11 ± 0.31	0.704
filling (f)		0.12 ± 0.44	0.76 ± 1.62	**0.025**
**dmft**		2.64 ± 2.51	3.39 ± 3.47	0.321
Decay (D)		0.96 ± 1.21	1.05 ± 1.47	0.794
Missing (M)		0 (0)	0.03 ± 0.16	0.422
Filling (F)		0.08 ± 0.4	0.21 ± 0.58	0.329
**DMFT**		1.04 ± 1.34	1.29 ± 1.51	0.504
PI (Positive)		9 (36)	16 (42.1)	0.793
BI (Positive)		2 (8)	0 (0)	0.154

*Chi-squared or student’s t-test as appropriate. DMFT and dmft stand for decayed, missing, filled teeth, for permanent and primary dentitions, respectively

### Sequencing and data preprocessing statistics

A total of 3,385,775 raw paired-end reads were obtained and deposited in the Sequence Read Archive (Project ID PRJNA691555). About 92% of the reads were successfully merged with PEAR, of which ~52% were filtered out at the quality-filtration step, and an additional 11% at the chimera check step. About 83% of the remaining sequences were successfully classified to the species-level (mean of 14,943 ± 5,315 reads per sample). The detailed sequencing and data preprocessing statistics are provided in Supplementary Dataset 1.

### General microbiological findings

**Figure 1.**
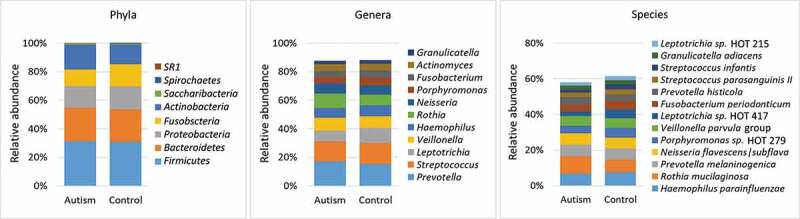
Major taxa identified in the study groups. Sequencing of the V1-V3 region of the 16S rRNA (2 x 300 bp) was performed on DNA extracted from tongue scraping samples. The raw sequences were merged, quality-controlled and assigned species-level taxonomies. The stacked bars show the average relative abundances of all phyla, top 11 genera and top 13 species (top taxa are those with average abundance of ≥ 2% in the control group). HOT: human oral taxon

Overall, 193 bacteria species, belonging to 51 genera and eight phyla were detected. The detection frequencies and relative abundances for each taxon in each study subject are presented in Supplementary Datasets 2–4. The number of taxa identified per subject ranged from 56 to 170 species (132 on average) and from 20 to 47 genera (39 on average). Fifty-five species and 25 genera were core taxa identified in at least 90% of the study subjects (Supplementary Datasets 5). The average microbial profile for each of the study groups is presented in Figure 1. At the phylum level, *Firmicutes, Actinobacteria, Proteobacteria, Fusobacteria*, and *Bacteroidetes* accounted for ~ 99% of the sequences in both groups. At the lower taxonomic levels, and for both groups, the top 11 genera (average abundance of ≥ 2% in the control group) accounted for nearly 90% of the average microbiome, while the top 13 species (average abundance of ≥ 2% in the control group) constituted ~ 60% of the reads. On average, *Prevotella, Streptococcus* and *Leptotrichia* were the most abundant genera, while *Haemophilus parainfluenzae, Rothia mucilaginosa* and *Prevotella melaninogenica* were the most abundant species.

### Richness, diversity and differential abundance

**Figure 2.**
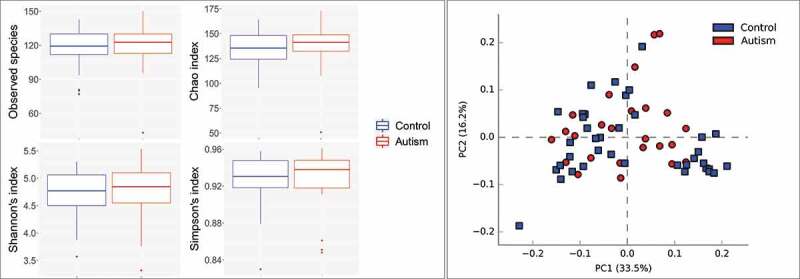
Species richness and diversity. Standard QIIME scripts microbiological profiles were subsampled and used to calculate observed richness, expected richness (Chao index), and alpha diversity indices (Shannon’s and Simpson’s). **Left**: Box and whisker plots of species richness and alpha diversity in each group. Differences were not significant by Mann–Whitney U test. Plots were generated with R Package. **Right**: clustering of samples with PCA using STAMP

**Figure 3.**
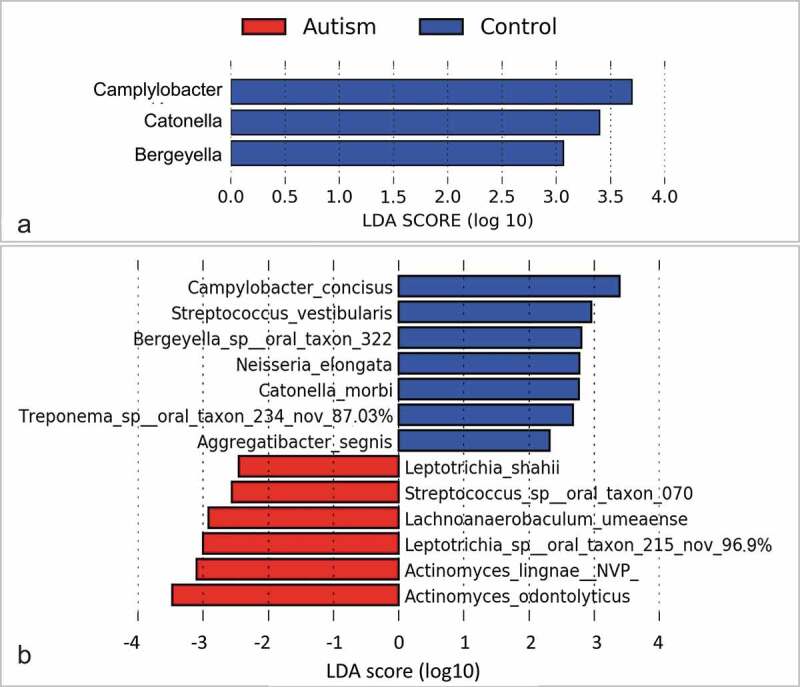
Differentially abundant taxa. **(a**) Genera and (**b**) species identified by linear discriminant analysis (LDA) effect size analysis (LEfSe) to differ in relative abundance between the two study groups as. None of them achieved a false discovery rate (FDR) ≤ 0.2. Nov: potentially novel species with the percentage indicating similarity to the closest hit

No significant differences were identified between the study groups in species richness, alpha diversity and beta diversity (Figure 2). By LEfSe analysis, 13 species and three genera were found to be differentially abundant between the two groups (Figure 3). For example, the tongue microbiome of the autism subjects had higher abundance of *Actinomyces odontolyticus, Actinomyces lingnae* and a potentially novel species with 97% similarity to *Leptotrichia* oral taxon 215, while lower abundance of *Campylobacter concisus, Streptococcus vestibularis* and *Bergeyella* oral taxon 322. However, none of the differences achieved an FDR ≤ 0.2.

Interestingly, comparison by gender revealed more significant differences with, five species and two genera identified as differentially abundant at an FDR ≤ 0.1 (Supplementary Figures 1–3).

## Discussion

To the best of our knowledge, this study is the first that shed light into the tongue microbiome of children with ASD in comparison to the healthy control peers. ASD is a neurological and developmental disorder of uncertain etiopathogenesis. Recent research suggests that the gut microbiome [9–13], and to lesser extent, the oral microbiome [22–24] may play a role. As far as the latter is concerned, studies have been limited to the use of saliva which is site non-specific, i.e. it contains a pool of microorganisms from tooth and mucosal surfaces, dental cavities, gingival sulcus, and periodontal pockets. The current study sought to assess the tongue microbiome instead, which represents a unique microbial environment [41,42]. To minimize contamination with saliva, we made utmost efforts to dry the tongue surface prior to sample collection. Dental health of ASD and healthy control were comparable except for more filled primary teeth among ASD, which minimized the confounding effect of the oral health status on the composition of the microbiome.

There were no significant differences between the two groups in species richness and alpha diversity, which is consistent with previous studies that assessed the salivary microbiome in ASD [22–24]. We also found that the two groups did not differ in beta diversity (PCoA analysis), which is similar to the findings reported by two previous studies [22,23]. Qiao et. al. [24], however, did demonstrate a significant separation between ASD and healthy children by PCoA, for both saliva and dental plaque. In this study, no differentially abundant features (genera and species) were identified between the two groups at FDR ≤ 0.2, which is quite consistent with the study by Kong et. al. [22] who only identified an unspecified *Bacillus* genus to be associated with ASD at FDR ≤ 0.2. Hicks et al. [23] identified 10 differentially abundant species between ASD and the healthy controls at FDR ≤ 0.1; however, with the exception of *Porphyromonas gingivalis*, all of these species were environmental rather than oral taxa (e.g. species belonging to *Planctomycetes, Cyanobacteria* and *Calditrichaeota*) suggesting contamination. Qiao et. al. [24] found 27 genera to be differentially abundant in the saliva of ASD at FDR ≤ 0.05, prominantly enrichment of *Haemophilus* and depletion of *Porphyromonas* and *Actinomyces*. The only similarity with our results at the nominal significance level is the depletion of *Catonella* in ASD.

The tongue microbiome has been scarcely assessed and in contexts other than autism and not involving children [25,27,43,44]. Hence, comparing our results against others will be limited to healthy adult samples. At the phylum level, our results are identical to what we revealed previously among healthy adults [44], and almost similar to what was revealed by Seerangaiyan et al. except for tiny proportions of *Saccharobacteria, Spirochetes* and SR1 in our sample but not theirs, and somewhat high proportion of TM7 in theirs, but not in ours [25]. At the genus level, the similarity with the above two studies is almost perfect, although with noticeable differences in the relative abundance, in addition to absence of *Capnocytophaga* and *Atopopium* in our study. The difference widens noticeably at the species level. This can be ascribed to the age; research has shown that the microbiome in different body sites changes with age, and thus is proposed as predictors of the chronological age [45].

Interestingly, comparison by gender revealed significant differences with five species and two genera identified as differentially abundant at an FDR ≤ 0.1. A recent study on the oral microbiome of children did not report similar results [46]. In contrast, a recent study found that the bacterial richness in subgingival plaque differed by gender in adults with and without cognitive dysfunction [47]. The observed differences in the tongue microbiome by gender in our study warrant further investigation.

In conclusion, the tongue microbiome of children with ASD was not found to be significantly different from that of healthy control children, which is largely consistent with results from the literature. Nevertheless, large-scale, more powered studies employing functional approaches (metabolomics, metatranscriptomics) are warranted to explore this further.

## Supplementary Material

Supplemental Material
