# Supplementary figures and images for: Tongue microbiome in children with autism spectrum disorder
Source: J Oral Microbiol. 2021 Jun 22;13(1):1936434. doi: 10.1080/20002297.2021.1936434 (PMC8221129; doi:10.1080/20002297.2021.1936434)

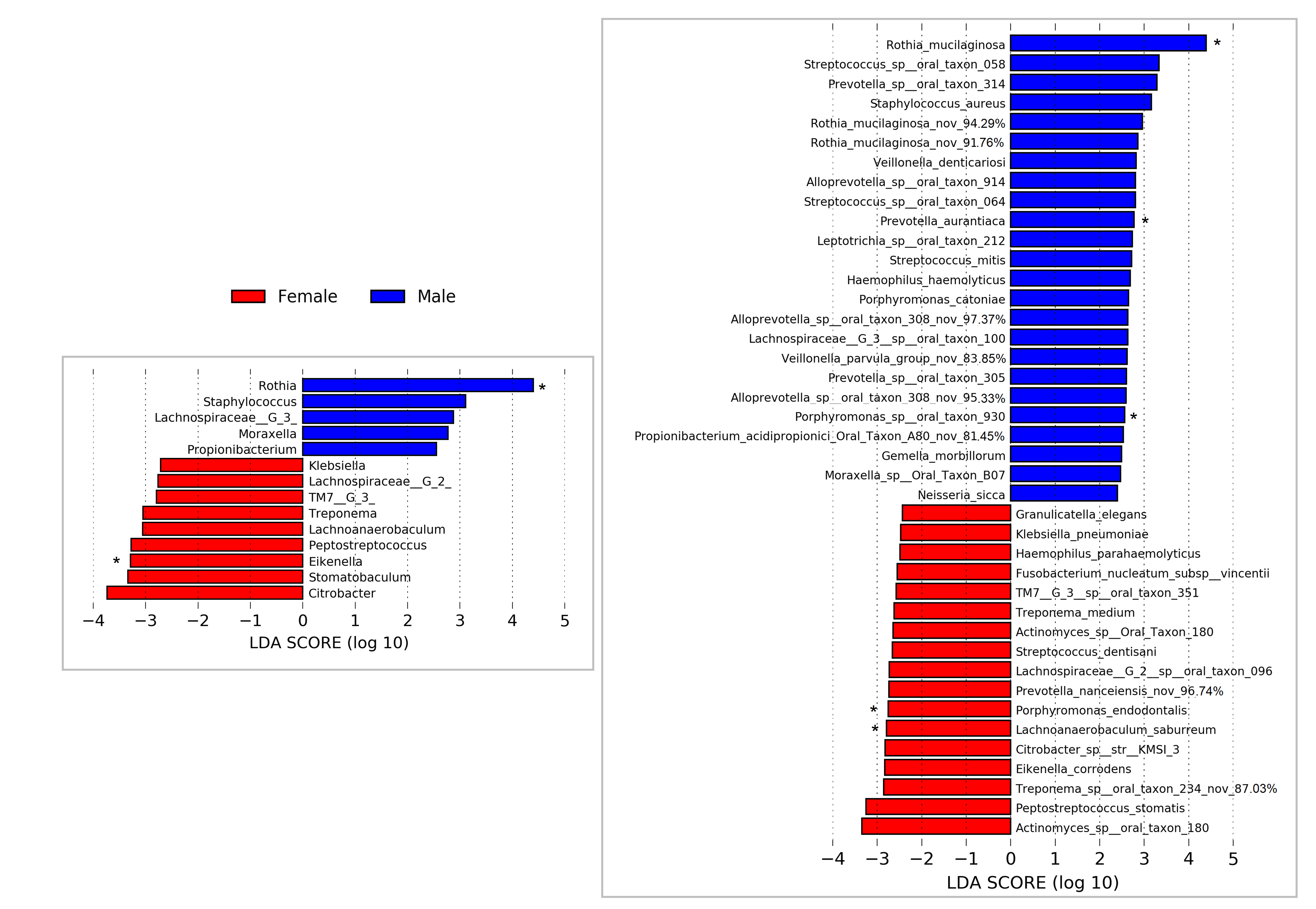

Supplement: Supplemental Material [file ZJOM_A_1936434_SM6948.zip › Supplementary/Supplementary figure 2_LEfSe analysis by gender.tif]

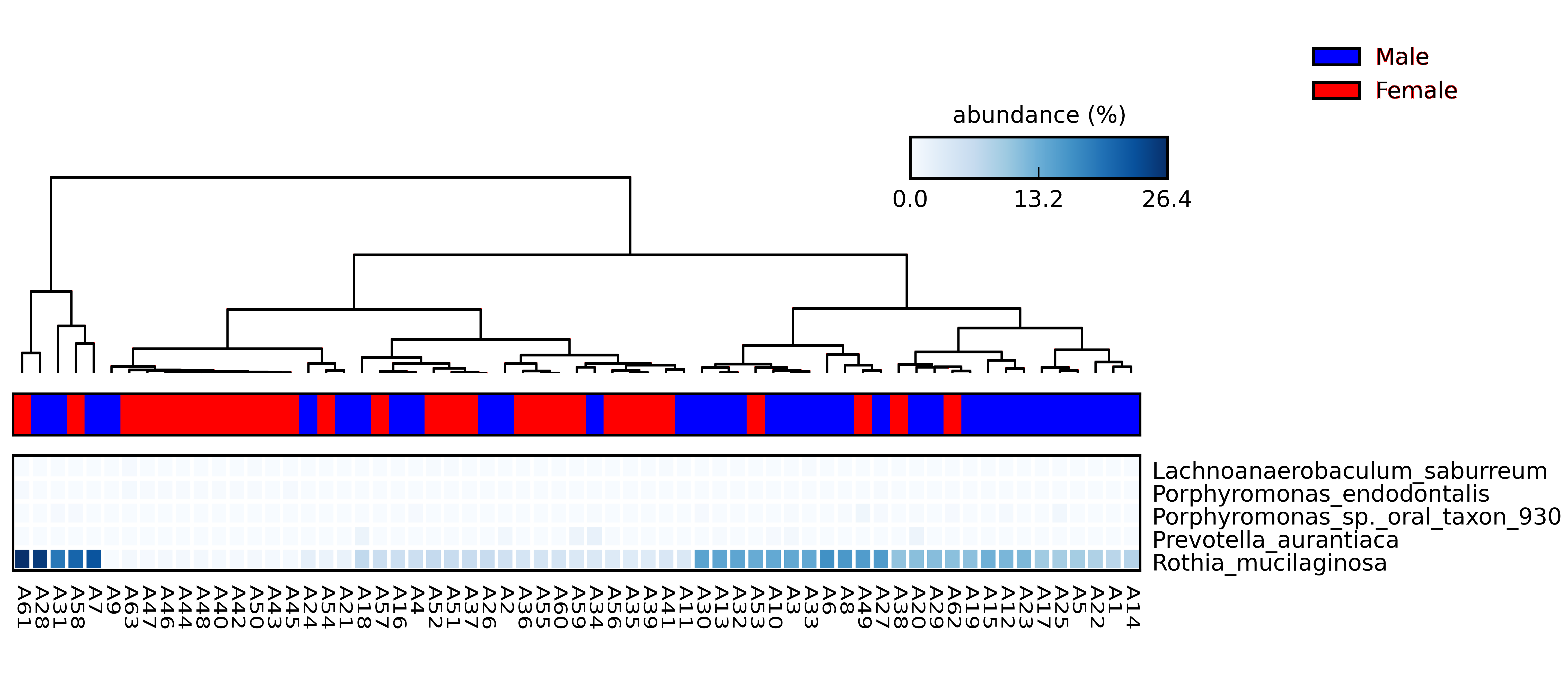

Supplement: Supplemental Material [file ZJOM_A_1936434_SM6948.zip › Supplementary/Supplementary Figure 3_Clustering by gender STAMP.tif]

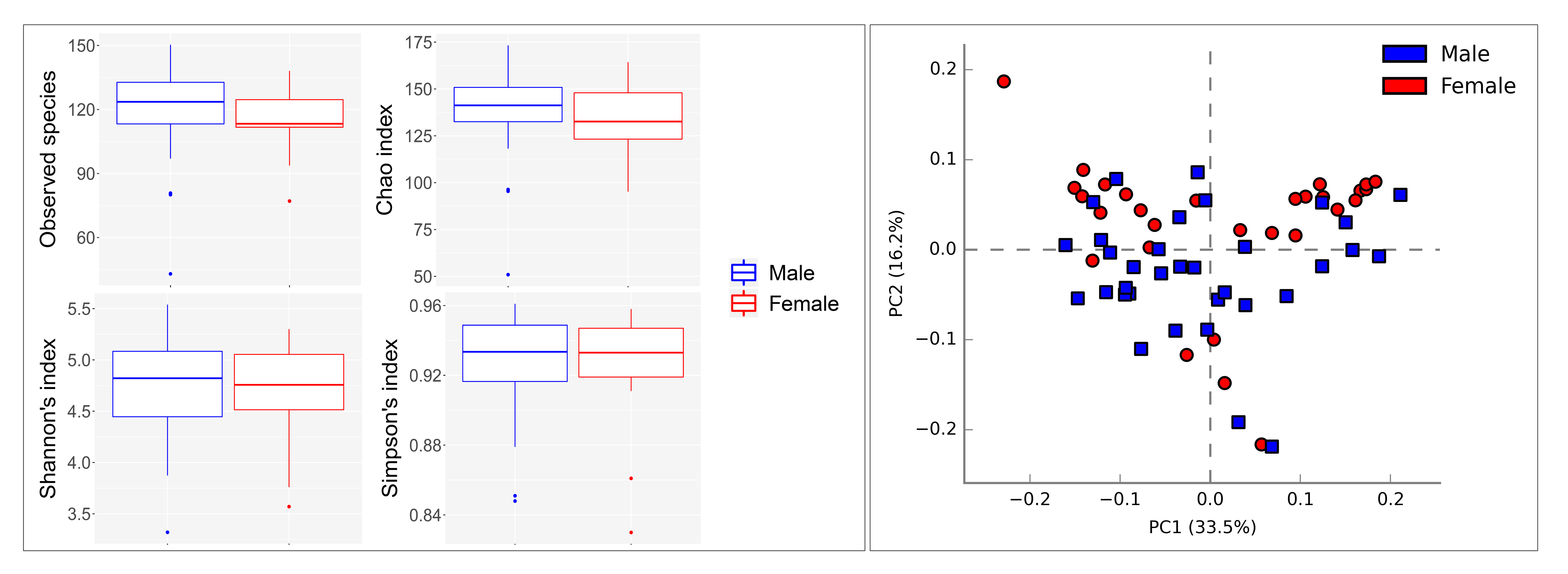

Supplement: Supplemental Material [file ZJOM_A_1936434_SM6948.zip › Supplementary/Supplemetary figure 1_Alpha diversity and PCoA_by gender.tif]
